# An evaluation of excellence in primary healthcare units after the introduction of a performance management innovation in two regional states of Ethiopia: a facility based comparative study

**DOI:** 10.1186/s12913-022-07885-8

**Published:** 2022-04-08

**Authors:** Wubishet Kebede Heyi, Elias Mamo Gurmamo, Amare Assefa Anara, Agegnehu Gebru Sendeku, Abera Refissa, Feyisa Serbessa Yadeta, Mesele Damte Argaw, Binyam Fekadu Desta

**Affiliations:** USAID Transform: Primary Health Care Project, JSI Research & Training Institute, Inc. in Ethiopia, P.O. Box 1392 code 1110, Addis Ababa, Ethiopia

**Keywords:** Performance management, Organizational culture, Primary healthcare unit, Excellence, Ethiopia

## Abstract

**Background:**

The Ethiopian Ministry of Health strives to achieve universal health coverage (UHC) through increasing the number of its high-performing primary healthcare units. Although the Ethiopian health system is managed within a decentralized political system, the Ministry of Health works towards institutionalizing performance management innovations and organizational cultures that increase the excellence of primary healthcare entities. To date, there has been little evidence gathered on the factors influencing the excellence of primary healthcare units in Ethiopia. Therefore, the aim of this study was to assess and compare how the introduction of performance management and organizational culture innovations through project support affect the excellence of primary healthcare units in Ethiopia.

**Methods:**

A facility-based comparative study was conducted in USAID Transform: Primary Health Care project supported and non-supported primary healthcare units located in the Oromia and Southern Nations Nationalities and Peoples’ (SNNP) regions of Ethiopia. Quantitative data were collected from randomly selected health workers using interviewer-administered questionnaires. In addition, primary healthcare unit excellence measurements were extracted from routine health information databases over eight quarters. The data were analyzed using the Statistical Package for Social Science (SPSS IBM v 20) research software package. Results were presented in frequency tables and graphs. After checking the data for homogeneous distribution, a paired sample t-test for equal variances, otherwise known as the Mann–Whitney U test was analyzed to claim statistically significant difference at *P* < 0.05.

**Results:**

Out of 368 invited health workers, 364 participated in this study, (a response rate of 98.9%). Slightly higher than two-thirds of participants were enrolled from the Jimma Zone of Oromia Region. Orientations on performance management standards were provided to 101 (68.2%) and 45 (48.3%) health workers from project-supported and non-supported facilities, respectively. The mean perceived organizational culture score with [± Standard Deviation (SD)] was 3.72 ± 0.75 among project-supported health workers and 3.385 ± 0.75 among non-supported health workers, respectively. An independent sample t-test showed statistically significant differences, where project-supported health workers had higher mean scores on perceived organizational culture than their non-supported counterparts, with t = 433, df = 362, *P* = 0.001. The mean baseline primary healthcare unit excellence score was 63.2% and 50.5% for project-supported and non-supported health facilities, respectively. The end line excellence scores increased to 93.3% for project-supported and 79.1% for non-supported facilities. The end line overall primary healthcare units’ mean rank excellence scores were 257.67 for the project supported and 105.66 for non-project supported facilities. This result of a non-parametric test, i.e. the Mann–Whitney U test revealed that project-supported facilities were higher and had a positive statistically significant difference (U = 2,728, z = -13.78, *P* = 0.001).

**Conclusions:**

The findings of this study underscore a direct relationship between implementing performance management innovations and enhancing organizational cultures for excellence at primary healthcare units. Project-supported primary healthcare units had higher organizational culture and excellence scores than their counterpart non-supported facilities. Therefore, achieving UHC through excellence in primary healthcare facilities requires scaling up of performance management innovation interventions.

**Supplementary Information:**

The online version contains supplementary material available at 10.1186/s12913-022-07885-8.

## Introduction

In the last two decades, the world has witnessed remarkable improvements in health —particularly in many low-income countries—towards ensuring availability of accessible, equitable, and quality essential healthcare to populations [1]. Despite these improvements, in low-income countries, a considerable number of mothers, neonates and children are dying due to preventable causes. Hence, as part of the Sustainable Development Goals (SDGs), 180 countries have committed to further reducing these deaths. According to the SDG pledges, by the year 2030, five of the seventeen SDGs will contribute to better health outcomes through strategic implementation of innovations that enhance good health and wellbeing for people, ensure quality education, expand decent work and economic growth, and further reduce inequalities [2].

The ability of a country to meet its goals depends largely on its existing performance management practices, organizational culture, and a high performing primary healthcare entity [3]. An organization’s culture defines the agreed norms within the organization. It is characterized by employees’ perceptions and behavior in an organization [4]. Well-functioning health leaders develop and shape subordinates through sharing values, beliefs, and assumptions [5]. Building organizational culture needs continuous effort and innovations [6].

The Ethiopian Health Sector Transformation Plan (2020- 2025) strategizes to achieve five major agendas, namely: quality and equity, information revolution, motivated competent and compassionate health workforce, health financing, and leadership [5]. The Ministry of Health implements the Ethiopian Health Center Reform Implementation Guidelines (EHCRIGs), as minimum standards that categorize primary healthcare unit excellence [7]. EHCRIGs are expected to be implemented in all health centers in Ethiopia. Development partners also provide technical and financial support through promoting commitment, responsiveness, and accountability at the primary healthcare level.

The USAID funded Transform: Primary Health Care is a bilateral project implemented with the Ethiopian Ministry of Health and its line structures. This six-year (2017—2022) project aims to contribute to national efforts of preventing child and maternal deaths (PCMDs), through supporting and implementing strategic initiatives. The project has set long term goals to achieve four high-level result outcomes which consist of: (1) improved management and performance of health systems, (2) increased sustainable quality of service delivery across the primary health care units’ (PHCUs’) continuum of care, (3) improved household and community health practices and health seeking behavior, and (4) enhanced program learning and impact policy and programming related to PCMDs. The project has supported 420 out of 1081 districts in Ethiopia [8].

The USAID Transform: Primary Health Care project institutionalizes performance management innovations at community, PHCU, and district levels of the health system [8]. It strives to improve the work environment and management systems through building visible leadership, enhancing culture of quality, availing client focused services, and exercising transparency and accountability in the health system. The project supports the health system on the cyclic implementation of performance management initiatives within primary healthcare entities. In addition—placing performance management dimensions at the center—its technical support has four clustered components which consist of: (1) introducing minimum standards, (2) performance measurement, (3) developing quality and performance improvement projects, and (4) reporting on progress. This is because performance management innovations help the health system enhance organizational culture and achieve organizational excellence [9].

There is empirical evidence of the influence of organizational culture and its four traits: involvement, consistency, adaptability, and mission, on higher performances of several business entities [3, 6, 10]. Currently, there is no clear information on the contribution of performance management innovations on building organizational culture and primary healthcare unit excellence in Ethiopia. Therefore, this study aimed to compare the influence of performance management innovations on building organizational culture and primary healthcare unit excellence in Ethiopia.

### Operational definitions

#### Involvement

Is an internal organizational factor in developing organizational culture and is signified by transparent communication, employee-focused leadership, and strong interpersonal relationships within the organization [10].

#### Consistency

Is an internal organizational factor in developing organizational culture and refers to the level of cohesion, integration, or agreement around values and norms [10].

#### Adaptation

Is an external factor in maintaining an organizational culture that reflects an evolutionary approach to organizational culture and suggests that cultures develop and persist because they help an organization to survive and flourish [10].

#### Mission

Is an external factor in maintaining an organizational culture that provides purpose and meaning by defining a social role and external goals for the organization [10].

#### Performance management innovation

Includes team-based training of health workers on minimum standards, use of data for decision making, and strategic problem-solving tools. In addition, the project assigned a health system strengthening expert, who provided technical support on continuous measurement of performances, development of do-able projects, and organization of experience sharing events and performance review meetings [4, 9].

## Methods and materials

### Study setting

In the last three decades, Ethiopia has endorsed a health policy, with the principles of decentralization and devolution of power to the district level [11]. A district is formed with a population of 60,000 to 100,000—empowered to manage sector offices including four to five primary healthcare units. The primary level of care consists of health centers that serve 15,000 to 25,000 people and which manage, on average, five satellite health posts, each serving 3,500 to 5,000 people [5]. A PHCU is led by health professionals with a minimum qualification of a Bachelor of Sciences in public health or nursing (2), general practitioner (optional), midwives (3), nurses (5), laboratory technicians or technologists (2), pharmacy technicians or pharmacists (3), ophthalmic nurse (1), psychiatric nurse (1), environmental health professional (1), cleaners (5), archive staff (6), a maintenance professional (1), morgue attendant (1), and health extension workers (10) [5].

USAID Transform: Primary Health Care has been supporting 1,843 health centers under 443 districts located in five regions, namely: *Tigray, Amhara, Oromia, Sidama*, and SNNP. *Jimma* is an administrative zone within the *Oromia* Region and is located in the southwest, 360 km away from Addis Ababa, the capital city of Ethiopia. The zone has a population of 3,598,359, living in 22 districts with 104 PHCUs. Out of these, 11 districts and 72 primary healthcare units have been receiving performance management innovation support. Similarly, *Kembata Tembaro* Zone has a population of 969,160 living in 11 districts with 31 PHCUs. Of these, five districts and 17 primary healthcare units have been receiving innovative performance management interventions [8].

### Study design and period

A facility-based comparative study was conducted to investigate PHCUs’ excellence between project-supported and non-supported facilities after implementing performance management innovation interventions for about two years. This study was conducted from 25 February 2021 to 30 April 2021.

### Analytical framework

**Fig. 1:**
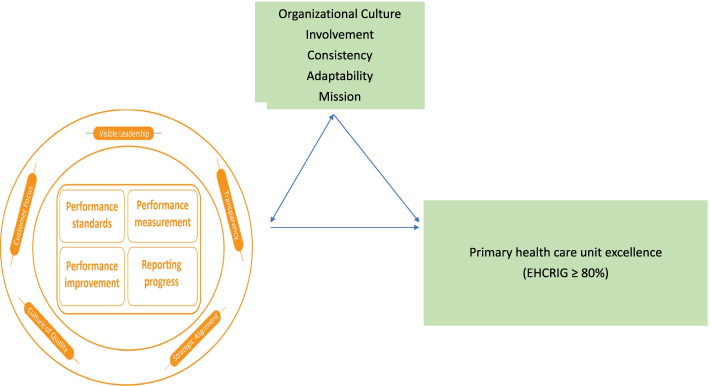
A conceptual framework developed to assess culture of excellence at PHCUs [10]. A modified conceptual map developed to evaluate performance management innovations in two zones of Ethiopia. The framework illustrates the relationship of performance management, organizational culture, and PHCU excellence

For this study, a modified conceptual model was adopted from the Public Health Foundation’s performance management and Denison’s organizational culture frameworks (Fig. 1) [10, 12]. The framework assumes that good performance management practices can lead to building an organizational culture. According to the Human Factor inc., (2009) a structured performance management process can strongly support ongoing efforts to build accountability into organizational culture [13]. It also helps keep everyone aligned with shared organizational goals, and focuses people’s attention on top organizational priorities. Similarly, Den Hartog et al., (2004) characterized performance management as managements’ day-to-day support and development of their staff [14]. Organizational culture was assessed using its four dimensions, namely: involvement, consistency, adaptability, and mission [10]. In addition, organizational excellence is the result of an organization’s culture and performance management practices [7]. To show the relationship among performance management innovations, organizational culture, and primary healthcare unit excellence, a logical flow of events best fits the process of descriptive and inferential statistical analysis. The first and second steps measured the perceived performance management innovation activities and traits of organizational culture through the participation of staff. The third step is to extract and transform organizational excellence measurement data, to categorize primary healthcare entities into three, namely: low-, medium-, and high-performance statuses.

### Source population and sampled population

The first target population for this study was all PHCUs located in Jimma and Kembata Temabro administrative zones of Ethiopia. The second target population was the health workforce serving in PHCUs. The sampled health workers’ educational backgrounds included health officers, nurses, midwives, laboratory technologists, pharmacists, and business management.

### Variables under study

#### Dependent variable

The overall score of PHCU excellence (the average scores of 10 chapters, 87 standards, and 209 validation criteria of EHCRIGs). Rated from 0.0% to 100.0%.

#### Independent variables

Performance management and organizational culture scores.

### Sample size and sampling procedures

The required sample size for this study was estimated by using a double population proportion formula:

*n* = (Zα/2 + Zβ)^2^ * (p1(1-p1) + p2(1-p2)) / (p1-p2)^2^. Where, Zα/2 is the critical value of the normal distribution at α/2 (i.e. confidence level of 95%, α is 0.05 and the critical value is 1.96), Zβ is the critical value of the normal distribution at β (i.e. for a power of 80%, β is 0.2 and the critical value is 0.84), p1 is 50% of PHCU excellence scores for non-project supported entities, and p2 is 65% for the project supported PHCUs. The level of statistical significance is 0.05, β = type II error 0.2, and the non-response rate, 10%. In this study, a total of 368 primary healthcare workers, that is, 184 from project-supported and 184 from non-supported PHCUs were sampled. The USAID Transform: Primary Health Care project implemented performance management innovations in Jimma and Kembta Tembaro administrative zones. Therefore, both zones were selected purposefully. However, research participants were enrolled using systematic random sampling methods.

### Data collection tools, procedure, and data quality assurance methods

Several tools were used to collect data required to address the objectives of the study. A structured questionnaire was employed to collect data that measured performance management practices and organizational culture. In addition, pretested data abstraction forms were used to collect PHCU excellence scores from a routine health information system database.

### Data quality assurance methods

As part of ensuring the quality of collected data, the investigators adopted pretested organizational culture [10], performance management, and PHCU excellence measurement tools [7]. Inputs were gathered from other similar studies (Additional file 1) [15, 16]. In addition, the data collectors were six public health officers who were trained and deployed to collect the required data in 38 PHCUs. The data collectors were identified, trained, and assigned to collect the necessary information by their respective zone health departments. Completeness and consistency of information were checked on the day of the data collection.

### Data analysis

The data was entered and analyzed using SPSS version 25 [17]. A descriptive analysis was used to present the summary of the data using graphs and frequency tables. To determine the magnitude of performance management using its four parameters, (i.e., minimum standards, measurements, quality and performance improvements, and collaborative functioning), 24 close-ended questions were scored with a “1” for positive and a “0” for a negative response (additional file 2). The overall performance management score was rated out of 24. To check the presence of statistical differences for categorical variables, a chi-square test was analyzed and a *P* < 0.05 was the decision criteria.

To estimate the organizational culture, 34 five-point Likert scale questions were rated by the participants (additional file 2). The organizational culture assessment tool has four sub-categories, namely: involvement (9 questions), consistency (8 questions), adaptability (8 questions), and mission (9 questions). The reliability test was determined using Cronbach’s alpha values and the results were 0.900 for involvement, 0.716 for consistency, 0.865 for adaptability, 0.916 for the mission, and 0.946 for the overall organizational culture. Therefore, the tool used to demonstrate organizational culture was consistent and reliable. An independent sample t-test was analyzed for homogeneous (Levene’s Test > 0.05) continuous variables and statistical significance was claimed at *P* < 0.05.

Excellence at PHCUs was assessed using minimum standards of EHCRIGs [7, 15, 16] which has10 chapters and 87 sets of minimum standards with 209 validation criteria (additional file 3). The tools were used to assess the availability of essential resources and services. The checklist used consists of ten chapters: (1) leadership and governance, (2) health center and health post linkage, (3) patient flow and service organization, (4) medical records management, (5) pharmacy services, (6) laboratory services, (7) clean, and safe health facility, (8) medical equipment management and biomedical engineering, (9) human resource management, and (10) quality improvement and health information system [7]. PHCU excellence was measured out of 100 percentage points and categorized into three: high performing PHCU (excellence) for scores greater or equal to 80.0%, medium performing for scores between 60.0% and 79.9%, and low performing for scores less than 60.0% [7]. The two-sample PHCU excellence measurement data violate the assumptions of a parametric test (Levene’s test < 0.05). The investigators compared the mean rank differences between project-supported and non-supported facilities using a non-parametric statistical test called the Mann–Whitney U test.

## Ethical considerations

This study was granted an ethical clearance from the JSI Research & Training Institute, Inc. institutional review board (IRB) ref number IRB #21-06E. Permission to conduct individual interviews was sought from facility managers. Informed written individual consent was obtained from each study participant. The investigators maintained national and international ethical principles, including ensuring anonymity and confidentiality.

## Results

Table 1 depicts the socio-demographic characteristics of study participants. The questionnaires were completed by 184 (100.0%) and 180 (97.8%) of participants recruited from project-supported and non-supported PHCUs, respectively. Thus, the overall response rate of this study was 364 (98.9%). Slightly higher than two-thirds of the study participants were enrolled from within the Oromia Region and three-fourth of the health workers were enrolled from rural PHCUs. The descriptive analysis ensured that project-supported and non-supported health workers had no statistically different distribution (*P* > 0.05).

**Table 1:** Socio-demographic characteristics of the research participants, August 2021

**Variable**	**Category**	**Implementation of Health Center Reform Guidelines**					**Total *N* = 364**	
		**Project-supported (*N* = 184)**		**Non-project-supported (*N* = 180)**		***P*-value**		
		**Frequency**	**Percent**	**Frequency**	**Percent**		**Frequency**	**Percent**
**Region**	Oromia	125	67.9%	120	66.7%	0.797	245	67.3%
	SNNP	59	32.1%	60	33.3%		119	32.7%
**Sex**	Male	121	65.8%	124	68.9%	0.525	245	67.0%
	Female	63	34.2%	56	31.1%		119	33.0%
**Address**	Rural	133	72.3%	141	78.3%	0.181	274	75.0%
	Urban	51	27.7%	39	21.7%		90	25.0%
**Age**	< 26 years	48	26.0%	54	30.0%	0.453	102	28.0%
	26 – 30 years	91	49.5%	74	41.1%		165	45.3%
	31 – 35 years	31	16.8%	37	20.6%		68	18.7%
	36 + years	14	7.6%	15	8.3%		29	8.0%
**Profession**	BSc public health officer	47	25.5%	34	18.9%	0.672	81	22.3%
	Diploma nurse	44	23.9%	46	25.6%		90	24.7%
	BSc nurse	23	12.5%	33	18.3%		56	15.4%
	Management (diploma and BA)	21	11.4%	25	13.9%		46	12.6
	Midwife (BSc and diploma)	19	10.3%	16	8.9%		35	9.6%
	BSc pharmacy	13	7.0%	12	6.7%		25	6.8%
	BSc laboratory technologist	10	5.4%	9	5.0%		19	5.2%
	Health information technician	7	3.8%	5	2.8%		12	3.3%
**Marital status**	Single	99	53.8%	92	51.1%	0.876	191	52.7%
	Married	84	45.7%	87	48.3%		171	46.9%
	Separated /divorced	1	0.5%	1	0.6%		2	0.5%
**Years of service**	< 6 years	81	44.0%	80	44.4%	0.619	161	44.2%
	6—15 years	100	54.4%	98	54.5%		198	54.4%
	16 + years	3	1.6%	2	1.1%		5	1.4%

### Performance management

Table 2 presents performance management practices using four major categories. Orientations on the minimum PHCU excellence standards (EHCRIGs) were provided to staff working in 101 (68.2%) project supported and 45 (48.3%) and non-project supported facilities. 98 (71.5%) of staff from project-supported facilities participated in the measurement of governance and management-related minimum standards, while 70 (60.9%) of staff from non-project-supported facilities were actively engaged in governance and management standard measurements.

**Table 2:** Performance management practices of project and non-project supported facilities, August 2021

Variable	Category	Performance management				X^2^	*P*-value
		**Project supported (*N* = 184)**		**Non-project supported (*N* = 180)**			
		N	%	N	%		
**Performance standard (n1 = 148 & n2 = 116)**	Onsite and off-site training	89	60.1%	42	36.21%	14.89	0.001
	Orientation in staff meetings	101	68.2%	56	48.3%	10.75	0.001
	Self-study (reading guidelines)	50	33.8%	44	37.9%	0.48	0.485
	Self-study (reading posters & job aids)	45	30.4%	52	44.8%	5.82	0.016
**Performance Measurement (n1 = 137 & n2 = 115)**	Governance/management	98	71.5%	70	60.9%	3.19	0.074
	Drugs and therapeutics	68	49.6%	38	33.0%	7.06	0.008
	Clean and safe health facility	66	48.2%	52	45.2%	0.22	0.639
	Infection prevention	49	35.8%	34	29.6%	1.08	0.297
	Quality/performance management	51	37.2%	31	27.0%	3.00	0.083
	Medical equipment management	35	25.5%	20	17.4%	2.43	0.118
**Performance improvement (n1 = 184 & n2 = 180)**	Antenatal care	103	56.0%	80	44.4%	4.84	0.028
	Skilled birth attendance	102	55.4%	85	47.2%	2.45	0.117
	Family planning	98	53.3%	78	43.3%	3.59	0.058
	Postnatal care	96	52.2%	67	37.2%	8.22	0.004
	Growth monitoring	88	47.8%	58	32.2%	9.22	0.002
	Clean and safe health facility	89	48.4%	55	30.6%	12.07	0.001
	Community-based health insurance	85	46.2%	52	28.9%	11.61	0.001
	Health center-health post linkage	71	38.6%	50	27.8%	4.79	0.029
	Laboratory services	70	38.0%	40	22.2%	10.8	0.001
	Pharmacy services	68	37.0%	39	21.7%	10.24	0.001
	Health information management	13	7.1%	47	26.1%	23.97	0.001
**Reporting progress**	Reviewed reports	150	81.5%	115	63.9%	23.32	0.001
	Data used for performance appraisals	115	62.5%	75	41.7%	15,82	0.001
	Experiences shared through alliance for quality	104	56.5%	64	35.6%	16.09	0.001

#### Perceived organizational culture

The mean overall perceived organizational culture score with [± Standard Deviation (SD)] was 3.72 ± 0.75 and 3.38 ± 0.7, for respondents from project and non-project supported facilities, respectively. After ensuring the assumption of homogeneity of the data set using Levene’s Test > 0.05, an independent sample t-test was analyzed. The organizational culture score was statistically higher among respondents working in project-supported rather than non-project-supported facilities, at t = 433, df = 362, *p* = 0.001 (Table 3).

**Table 3:** Comparing average organization culture scores against project support status, August 2021

Indicators	Project supported (*N* = 184)		Non-project supported (*N* = 180)		t-test	*P*-value
	**Mean**	**SD**	**Mean**	**SD**		
**Empowerment**	3.74	1.01	3.30	1.05	4.04	0.001
**Team orientation**	3.79	0.89	3.46	0.88	3.50	0.001
**Capability development**	3.76	0.91	3.41	1.03	3.43	0.001
**Involvement**	**3.76**	**0.82**	**3.39**	**0.82**	**4.32**	**0.001**
**Core values**	3.77	0.83	3.51	0.93	2.80	0.001
**Agreement**	3.66	0.94	3.23	0.97	4.23	0.001
**Coordination and** i**ntegration**	3.78	1.83	3.15	1.09	3.92	0.001
**Consistency**	**3.75**	**1.00**	**3.31**	**0.85**	**4.47**	**0.001**
**Creating change**	3.57	0.95	3.30	1.09	2.52	0.012
**Customer focus**	3.69	0.89	3.37	0.96	3.30	0.001
**Organizational learning**	3.54	0.96	3.24	0.87	3.09	0.002
**Adaptability**	**3.40**	**0.82**	**3.30**	**0.82**	**3.44**	**0.001**
**Strategic direction and intent**	3.83	0.83	3.58	0.90	2.74	0.006
**Goals and objectives**	3.67	0.89	3.48	0.83	2.11	0.035
**Vision**	3.76	0.88	3.48	0.92	2.95	0.003
**Mission**	**3.75**	**0.80**	**3.51**	**0.77**	**2.93**	**0.004**
**Overall organizational culture**	**3.72**	**0.75**	**3.38**	**0.71**	**4.33**	**0.001**

#### Excellence at PHCUs

**Fig. 2:**
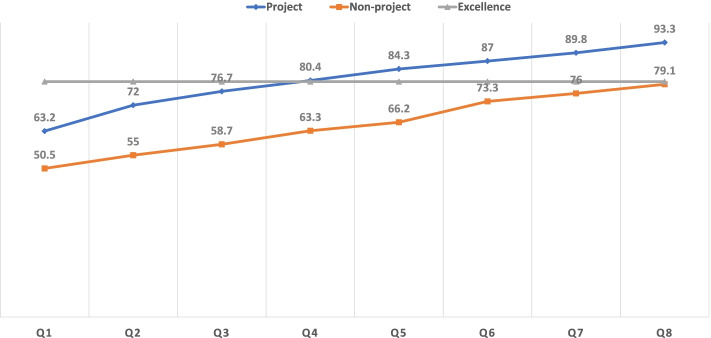
Trends in PHCU excellence scores by project support status, August 2021. Line chart showing the criteria for PHCU excellence, and trends of achievement of project and non-project supported health facilities. Results of a non- parametric statistical test which depicts the relationship of EHCRIG mean rank scores by project support status

Table 4 presents quarterly excellence scores of PHCUs in three categories. At the first measurement, 1 (5.0%) project-supported facility had achieved a score of excellence. This score shows an increasing trend, and all project-supported facilities achieved the measurement criteria at the seventh measurement. Conversely, 1 (5.5%) and 12 (66.6%) of facilities from non-project supported facilities achieved excellence scores against the criteria at fourth and eighth measurements, respectively. Figure 2 depicts trends in excellence scores of PHCUs against measurement criteria and status of project support. During the first measurement, the overall EHCRIG score was 63.2% and 50.5% for project and non-project supported facilities, respectively. The subsequent scores showed an increasing trend between both groups of facilities. At the fourth measurement, project-supported facilities achieved the minimum criteria for PHCU excellence (EHCRIGs score = 80.4%), while non-project-supported facilities achieved the minimum standards (EHCRIG score = 79.1%) at the eighth quarter of measurements.

**Table 4:** Excellence scores of PHCUs in three categories, August 2021

Excellenc ecriteria	QI		Q II		Q III		Q IV		Q V		Q VI			Q VII			Q VIII	
	P	NP	P	NP	P	NP	P	NP	P	NP	P	NP	P		NP	P		NP
< 60.0%	9	15	4	8	2	5	0	1	0	2	0	1	0		1	0		1
60.0%- 79.9%	10	3	8	10	8	13	9	15	5	15	4	13	0		9	0		5
80.0% +	1	0	8	0	10	0	11	2	15	1	16	4	20		8	20		12
Total	20	18	20	18	20	18	20	18	20	18	20	18	20		18	20		18

NB: *P* project supported, *NP* non-project supported

The data collected on PHCU excellence violates the assumption of the equality of variance, (Levene’s test *F* = 8.49, *P* < 0.004; *F* = 19.63, *P* < 0.001; *F* = 25.81, *P* < 0.001) on all three of EHCRIGs measurements. The investigators analyzed the mean rank scores between project and non-project supported facilities using a non-parametric test named the Mann–Whitney U test (Table 5). The results of the first measurements showed that the overall PHCU mean rank excellence score was 216.87 for project and 147.37 for non-project supported facilities (U = 10,236.50, z = -6.30, *P* = 0.001). Similarly, the results of the eighth measurements revealed statistically different overall PHCU excellence mean rank scores of 257.67 for project and 105.66 for non-project supported facilities (U = 2,728, z = -13.78, *P* = 0.001).

**Table 5:** PHCU excellence scores mean rank test using Mann–Whitney U Test, August 2021

	EHCRIG (Q1)		EHCRIG (Q4)		EHCRIG (Q8)	
	Project supported	Non-project supported	Project supported	Non-project supported	Project supported	Non-project supported
N	184	180	184	180	184	180
Mean rank	216.87	147.37	244.68	118.93	257.67	105.66
Sum of ranks	39,903.50	26,526.50	45,022.00	21,408.00	47,412.00	19,018.00
Mann–Whitney U	10,236.50		5118.00		2728.00	
Wilcoxon W	22,940.00		21,408.00		191,018.00	
Z	-6.30		-11.40		-13.78	
Asymp. sig. (2-tailed)	0.001		0.001		0.001	

## Discussion

In the last three decades—as with many low-income countries—several health reforms have been adopted and widely implemented to improve access to and quality of essential health services in Ethiopia. Some of the reforms are innovated, piloted, and scaled up through technical and financial support of development partners. This comparative quantitative survey was conducted to investigate the relationship between performance management innovation and organizational culture, and excellence at primary healthcare entities in two administrative zones of Ethiopia. The study shows that the distribution of the study participants was not different by comparison group. In addition, USAID Transform: Primary Health Care project supported PHCUs managed to achieve the criteria for excellence in a much shorter span of time than non-supported facilities. The study elucidates evidence of increased high-performing PHCUs through implementing performance management innovations and through enhancing organizational culture.

The project innovates performance management to enhance PHCUs’ organizational culture. The parameters were introducing minimum standards, regular measurements, developing improvement plans, and organizing knowledge sharing events. These activities were implemented over eight independent quarters to build organizational culture. A structured performance management process has five cyclic steps. The first step is establishing goals for individuals, teams, and organizations through linkage with a strategic framework [13, 14]. The second step is to develop a prioritized plan to achieve organizational commitment. The third step is providing direction to garner commitment from staff. The fourth step is to monitor the status of committed activities and provide feedback to staff. The fifth step is recognizing individuals and team contributions for organizational success. This step-by-step process supports ongoing efforts to build transparency and accountability into the desired organizational culture [13, 14].

The performance management innovation that was implemented in the PHCUs is consistent with the recommended parameters of the Public Health Foundation [9]. The conceptual framework assumed the importance of exercising visible leadership in creating shared direction and sound work environments to mobilize and involve staff in reform initiatives. In this study, participants selected from project-supported facilities were more likely to be involved in creating high-performing PHCUs than their counterparts working in non-supported facilities. The results revealed a larger number of project-supported study participants engaged in performance improvement activities and in reporting on progress collaborative functions (*P* < 0.05) while equally participating in performance measurement activities (*P* > 0.05). This significant difference could be because of the direct expert support in the form of onsite coaching and mentoring, and regular follow-up of developed performance improvement projects. Furthermore, the adopted performance management innovation model assisted PHCU leaders to align routine activities with strategic priorities and improve the culture of quality at their facilities [9]. The performance management innovation enhanced the leadership competencies of a hybrid of clinical and management professionals working in PHCUs [18, 19]. The intervention was designed to build organizational culture along with key performance indicators for PHCU excellence. The project supported the institutionalization of organizational culture at PHCUs in Ethiopia. Enhancing organizational culture is believed to promote the strategic direction, mission, and values among internal and external stakeholders. The findings of this research showed that the perception score of participants recruited from performance management innovation-supported facilities had a higher organizational culture score with a statistically significant positive difference when compared with scores of participants recruited from non-supported facilities. This finding was in line with the Maithel et al., (2012) and Vainieri et al., (2019) reports on the success of an organization with opportunities to learn and communicate norms among staff having had a positive effect on employee motivation and high organizational performances [20, 21]. Many developing countries have adopted and implemented minimum standards to increase the number of well-functioning and high-performing PHCUs [22]. The main purpose of working against minimum standards at PHCUs is to strengthen the health system and achieve global commitments in a shorter amount of time [23]. According to the results of this study, there is a high and statistically significant difference between the proportion of high-performing health facilities and project performance management innovation implementation status. PHCUs achieved excellence after four and eight measurements for project-supported and non-supported facilities, respectively. This finding could be because of the close support of development partners.

## Conclusions

The findings of this facility-based comparative study underscore a direct relationship between implementing performance management innovations to enhance organizational culture and excellence at PHCUs. However, as an observational study, it has both strengths and limitations. The strengths of this study include targeting two regional states in a decentralized health system in Ethiopia. In addition, the study gave opportunity to investigators and program managers to compare the status of performance management, organizational culture, and PHCU excellence by project support status. Furthermore, the investigators collected and analyzed multiple sources of routine health information system data to estimate the level of PHCU excellence over eight quarters in Ethiopia. Nevertheless, this study also has methodological limitations to claim a causality relationship. Since performance management innovation and organizational culture scores were measured with interviewer-administered questionnaires, this might cause some form of social desirability bias. Performance management innovation supported facilities had higher organizational culture and PHCU excellence scores. Therefore, to achieve UHC through increased PHCU excellence, scaling-up of performance management innovation interventions is required.

## Supplementary Information

**Additional file1.** A structured interviewer administered a questionnaire to collect data on performance management and organizational culture.

**Additional file2.** Data file.

**Additional file3. **A structured checklist developed and endorsed by the Ministry of Health to measure PHCU excellence. 

## Data Availability

The datasets used and/or analyzed during the current study are included in the manuscript and attached as a supplementary file.

## Abbreviations

EHCRIGs: Ethiopian Health Center Reform Implementation Guidelines
SD: Standard deviation
SDGs: Sustainable Development Goals
SNNP: Southern Nations and Nationalities of Peoples’
SPSS: Statistical Package for Social Science
USAID: United States Agency for International Development
UHC: Universal health coverage
