# Supplementary material for: An evaluation of excellence in primary healthcare units after the introduction of a performance management innovation in two regional states of Ethiopia: a facility based comparative study
Source: BMC Health Serv Res. 2022 Apr 8;22:460. doi: 10.1186/s12913-022-07885-8 (PMC8991682; doi:10.1186/s12913-022-07885-8)
Supplement: Supplementary file 1 — Additional file1. A structured interviewer administered a questionnaire to collect data on performance management and organizational culture. [file 12913_2022_7885_MOESM1_ESM.docx]

| **Section A: - Personnel and Health Facility information** | | | |
| --- | --- | --- | --- |
| **SN** | **Question** | **Coding Categories** |  |
| **I** | **Part I: Basic Information** | | |
| 1.1 | Date of assessment | __ __/__ __/__ __ __ __(DD/MM/YYYY) |  |
| 1.2 | Region | 1. Oromia 2. SNNP |  |
| 1.3 | Organization name (code) | 1.2.1 District (Woreda)------------- 1.2.2. HC ------------------ |  |
| 1.5 | USAID Transform: PHC supported facility | 1. Yes 2. no |  |
| 1.6 | Location of the Health Facility | 1. Urban Center 2. Rural |  |
| 1.7 | Age [in Full Years] | [ ] years |  |
| 1.8 | Sex | 1. Male 2. Female |  |
| 1.9 | Marital status | 1. Single 2. Married 3. Separated/Divorced 4. Other (state)-------------------------- |  |
| 1.10 | Profession | 1. BSc Nurse 2. BSc Public Health Officer 3. Diploma Nurse 4. Midwifes 5. MSc in Health Service Management 6. Pharmacy 7. Laboratory 8. Support Staff 9. HIT 10. Other (Specify…………………………..) |  |
| 1.11 | Work experience in Year(s) | [ ] Years |  |
| 1.12 | Current position | [ ] specify |  |
| 1.13 | Have you heard of EHCRIGs? | 1. Yes 2. No |  |
| 1.14 | If your answer for Q1.13 is Yes, what was the source of your information | 1.10.1 Through training 1. Yes 2. No  1.10.2 Through department meeting 1. Yes 2. No  1.10.3 Through reading materials 1. Yes 2. No  1.10.4 Through posted materials 1. Yes 2. No  1.10.5 Through public medias (e,g, website) 1. Yes 2. No  1.10.6. Through association gatherings 1. Yes 2. No |  |

| **Section B: - Performance management practices** | | |
| --- | --- | --- |
| 1.15 | Did you participate in the implementation of EHCRIGs (Any participation during measurement and development of change idea for improvement) | 1. Yes 2. No |
| 1.16 | The performance standards (EHCRIGs), indicators, and targets are communicated to all healthcare workers (PS) | 1. Yes 2. No |
| 1.17 | The health facility measures most of the established individual/ department performance standards and targets (PM) | 1. Yes 2. No |
| 1.18 | The performance measurement reports are effectively used for decision making (PI) | 1. Yes 2. No |
| 1.19 | The health facility has regularly reported the performance of health workers in Ethiopian Primary Health Care Alliance for Quality (EPAQ) | 1. Yes 2. No |
| 1.20 | In the last twelve months, please tell us in how many EPAQ meetings you participated | [ ] number |

Please indicate the performance improvement projects developed, implemented, and monitored with your active participation at the health

| Ser no | Chapter’s description | Yes | No |
| --- | --- | --- | --- |
| 1.21 | Increase ANC4+ service uptake coverage | 1 | 2 |
| 1.22 | Increase skilled delivery coverage | 1 | 2 |
| 1.23 | Increase FP service coverage | 1 | 2 |
| 1.24 | Increase PNC service uptake coverage | 1 | 2 |
| 1.25 | Increase immunization service uptake & coverage | 1 | 2 |
| 1.26 | Increase growth monitoring coverage (6 – 59 months) |  |  |
| 1.27 | Increase CaSH (Clean and safe health facility) coverage | 1 | 2 |
| 1.28 | Increase Community-based health insurance (CBHI) coverage | 1 | 2 |
| 1.29 | Improves health center and health post linkages | 1 | 2 |
| 1.30 | Improves laboratory services | 1 | 2 |
| 1.31 | Improves pharmacy services | 1 | 2 |
| 1.32 | Improves medical record and RHIMs | 1 | 2 |
| 1.33 | Other (Specify) |  |  |
|  |  |  |  |

Please extract the health center reform achievements by chapters for eight quarters (Sept 2018- Sept 2020)

| Ser no | Chapter’s description | Percentage (%) | | | | | | | |
| --- | --- | --- | --- | --- | --- | --- | --- | --- | --- |
|  |  | Q1 | Q2 | Q3 | Q4 | Q5 | Q6 | Q7 | Q8 |
| 1.34 | Health Center Leadership, Management & Governance |  |  |  |  |  |  |  |  |
| 1.35 | Health center and Health post linkage |  |  |  |  |  |  |  |  |
| 1.36 | Patient Flow and Service Organization |  |  |  |  |  |  |  |  |
| 1.37 | Medical Record Management |  |  |  |  |  |  |  |  |
| 1.38 | Pharmacy Services Management |  |  |  |  |  |  |  |  |
| 1.39 | Laboratory Services Management |  |  |  |  |  |  |  |  |
| 1.40 | Safe and Clean Health Facility |  |  |  |  |  |  |  |  |
| 1.41 | Medical Equipment Management and Biomedical Engineering |  |  |  |  |  |  |  |  |
| 1.42 | Human Resource Management |  |  |  |  |  |  |  |  |
| 1.43 | Quality Improvement and Routine Health Information Management System Management |  |  |  |  |  |  |  |  |
| 1.44 | Overall HC Reform Achievements |  |  |  |  |  |  |  |  |

**Section C : culture of excellence at PHCU**

| SN | Dimensions | Indicators | Item description | Strongly Disagree | Disagree | Neutral | Agree | Strongly Agree |
| --- | --- | --- | --- | --- | --- | --- | --- | --- |
|  |  |  |  |  |  |  |  |  |
| IN1 | Involvement | Empowerment | Decision is taken based in enough information | 1 | 2 | 3 | 4 | 5 |
| IN2 |  |  | Information sharing to the employees | 1 | 2 | 3 | 4 | 5 |
| IN3 |  |  | Each employee believes in his/her positive role in the health facility | 1 | 2 | 3 | 4 | 5 |
| IN4 |  | Team Orientation | Awareness of teamwork | 1 | 2 | 3 | 4 | 5 |
| IN5 |  |  | The importance of supervision and horizontal coordination | 1 | 2 | 3 | 4 | 5 |
| IN6 |  |  | Team cohesiveness | 1 | 2 | 3 | 4 | 5 |
| IN7 |  | Capability development | Ability to develop compared to competitors | 1 | 2 | 3 | 4 | 5 |
| IN8 |  |  | the increase of employees' skill | 1 | 2 | 3 | 4 | 5 |
| IN9 |  |  | Development of employees' ability as an important source for health facility competitiveness | 1 | 2 | 3 | 4 | 5 |
| CN1 | Consistency | Core values | Leaders' knowledge of roles and responsibility | 1 | 2 | 3 | 4 | 5 |
| CN2 |  |  | All employees' grasp of organizational values | 1 | 2 | 3 | 4 | 5 |
| CN3 |  |  | Ethical code becomes guidance of common behavior | 1 | 2 | 3 | 4 | 5 |
| CN4 |  | Agreement | Common awareness to find solution of every organization | 1 | 2 | 3 | 4 | 5 |
| CN5 |  |  | Easiness to reach consensus | 1 | 2 | 3 | 4 | 5 |
| CN6 |  | Coordination & integration | Employees from different units share information | 1 | 2 | 3 | 4 | 5 |
| CN7 |  |  | Work coordination or activity is easy to perform | 1 | 2 | 3 | 4 | 5 |
| CN8 |  |  | Harmony in purposes between organization levels runs well | 1 | 2 | 3 | 4 | 5 |
| AD1 | Adaptability | Creating change | Responsive and easy to make change | 1 | 2 | 3 | 4 | 5 |
| AD2 |  |  | Having good responses to competitors and environmental changes | 1 | 2 | 3 | 4 | 5 |
| AD3 |  |  | Continuously adopting new and better ways | 1 | 2 | 3 | 4 | 5 |
| AD4 |  | Customer focus | Customer’s evaluation and recommendation are applied for basic changes | 1 | 2 | 3 | 4 | 5 |
| AD5 |  |  | Consumers' suggestions directly affect decision | 1 | 2 | 3 | 4 | 5 |
| AD6 |  | Organizational learning | Failure is applied as consideration to a better direction | 1 | 2 | 3 | 4 | 5 |
| AD7 |  |  | Awarding risk-takers | 1 | 2 | 3 | 4 | 5 |
| AD8 |  |  | Action and work performance are carried out after coordination with concerned units | 1 | 2 | 3 | 4 | 5 |
| MS1 | Mission | Strategic direction & intent | Having goals and long-term directions | 1 | 2 | 3 | 4 | 5 |
| MS2 |  |  | Having clear missions as directions for work performance | 1 | 2 | 3 | 4 | 5 |
| MS3 |  |  | Having clear future strategies | 1 | 2 | 3 | 4 | 5 |
| MS4 |  | Goals & objectives | Organization goals are agreed by all people in the health facility | 1 | 2 | 3 | 4 | 5 |
| MS5 |  |  | Leaders set ambitious but realistic goals | 1 | 2 | 3 | 4 | 5 |
| MS6 |  |  | Leaders clearly clarify objectives to obtain | 1 | 2 | 3 | 4 | 5 |
| MS7 |  | Vision | Having shared vision on what that will be materialized | 1 | 2 | 3 | 4 | 5 |
| MS8 |  |  | Having long-term orientation | 1 | 2 | 3 | 4 | 5 |
| MS9 |  |  | Having vision of creating spirit and motivation among employees | 1 | 2 | 3 | 4 | 5 |

***THANK YOU FOR PARTICIPATING IN THE SURVEY.***
